# Artificial intelligence in the NHS: Moving from ideation to implementation

**DOI:** 10.1016/j.fhj.2024.100183

**Published:** 2024-09-19

**Authors:** Anmol Arora, Tom Lawton

**Affiliations:** a School of Clinical Medicine, University of Cambridge, Cambridge CB2 0SP, UK; b Department of Oncology, University College London, London WC1E 6DD, UK; c Department of Computer Science, University of York, York YO10 5GH, UK; d Improvement Academy, Bradford Teaching Hospitals NHS Foundation Trust, Bradford BD9 6RJ, UK

Artificial intelligence (AI) is conventionally defined as the ability of a computer system to perform tasks that are usually associated with human intelligence, such as learning, reasoning and self-correction.^1^ The moral imperative to improve patient care coupled with pre-existing large datasets has enabled healthcare as an industry to lead the development of frontier AI models. In imaging, AI has been able to detect features such as age, blood pressure, and cardiovascular risk that are indeterminable to human ophthalmologists from routinely collected fundus photos.^2^^,^^3^ AI large language models (LLMs) show potential in both diagnosis and selection of treatment plans.^4^ Indeed, potential use cases of AI in healthcare are broad and stretch far beyond direct facing patient care, with advancement in AI in drug discovery, in improving the quality of medical education and in even automation within a medical research setting.^5^

The UK National Health Service (NHS) benefits from high levels of public trust, existing data infrastructure and close collaboration between academic groups and hospital trusts. Compared to other systems, such as the USA, which exhibit more complex patient–provider relationships, it can be easier to align the relevant stakeholders of patients, providers, government and developers. However, as alluded to by Burns *et al*,^7^ there are active efforts to align these parties in the USA, including through the Healthcare AI Commitments Initiative that provides a harmonised framework towards AI innovation for industry.^6^^,^^7^ Burns *et al*^7^ highlight the role that industry has played in delivering clinical AI solutions and this is seen with two articles in this issue highlighting industry–NHS partnerships. Pope *et al*^8^ present a case study of a successful industry–NHS collaboration between Roche and Great Ormond Street Hospital.^8^ They describe the practicalities involved with sharing anonymised health data and the role of trusted research environments (TREs). Balloch *et al*^9^ present results from early testing of an ambient AI tool to automate note taking during medical consultations using a novel commercial tool.^9^

Misra *et al*^10^ provide an insight into the implications of clinical AI for medical education. It remains an open question as to how much doctors should know about the AI algorithms that they may be interpreting in the near future.^10^ If we accept that AI will be used in clinical decision making, it will be essential for doctors to have some understanding of the algorithmic processes underlying AI decision making. Adapting medical education to the digital age will take time and while there have been calls for further evolution of curricula to include digital skills, this remains absent from national guidance for what is expected from medical school graduates.^11^ Perhaps even more challenging than updating medical school curricula will be the need to launch educational programmes for the existing workforce. A training programme for doctors aimed to teach even the basic underpinnings of AI algorithms would be expensive, difficult to implement and even more difficult to monitor. Doctors and their employers have a high opportunity cost to engage in a formal training programme, with the potential for lost income, lost time and the predictable scheduling difficulties associated with busy clinical rotas. The topic of AI education remains a barrier to implementation from several perspectives discussed in this issue, including by Davies *et al*^12^ in the context of the potential environmental costs of AI education.^12^

Development of algorithms has at times outpaced regulation, though the international hype surrounding ChatGPT has triggered a sense of need for novel regulation. Fotheringham and Smith^13^ discuss the importance of regulatory intervention, also arguing that, while awaiting legislation, clinicians should at least be provided with professional guidance to enable AI systems to be used safely.^13^ The concept of who shoulders responsibility for an error made by an AI system is also surfaced in an article about the patient safety implications of clinical AI by Habli *et al.*^14^ who emphasise that the safety of a clinical AI system is dependent on the workflow it is deployed within rather than solely reliant upon its performance metrics. They also highlight the risk of ‘scope creep’, whereby AI devices initially approved for a restricted use-case may over time begin to be used for more complex tasks beyond their intended purpose. Crider^15^ draws the analogy of the autopilot being implemented to assist pilots, stating that the existence of the autopilot ‘numbed’ the critical thinking of pilots during its early introduction and unexpectedly led to an increase in crashes due to automation bias.^15^

Successful innovation in healthcare is typically characterised by the involvement of four key stakeholders: inventors, healthcare providers, regulators and the patient. Arguably, the most important voice is that of the patient, especially in a publicly funded health system where the government is directly accountable to patients and the public. Winfield^16^ highlights concerns from the public, including the risks of algorithmic bias and losing the human connection in healthcare and data privacy.^16^ Winfield's contribution to the issue highlights public sentiment that there is support for developing clinical AI systems, but that these must involve the patients and the public. Here, the UK benefits from a world-leading approach towards patient and public involvement (PPI) in research driven by organisations such as the National Institute for Health and Care Research (NIHR).

This theme of direct collaboration between academics, healthcare professionals and the public to produce AI solutions is discussed in more detail by Welsh *et al*.^17^ They suggest that AI innovation should challenge the orthodoxy of patients being considered recipients of a care service and instead should be recognised as co-creators, sharing both their data and insights when new algorithms are being developed. Similarly, they discuss that healthcare professionals must be actively involved in the design of novel systems, rather than simply being considered end-users of AI tools being produced by others. Carey *et al*^18^ argue that both clinicians and the AI systems themselves have a role in ensuring that AI use is fair, particularly when systems become more complex and difficult to unpick than examples like false oximetry readings with dark skin.^18^ The interaction between clinicians and AI systems is also discussed by McCradden and Stedman,^19^ who argue that clinicians should be able to triangulate sources of evidence to inform their clinical reasoning rather than solely relying on the output of a clinical AI algorithm, especially when there is questionable explainability of such algorithms.^19^

This special issue intentionally encompasses a broad range of perspectives to provide what we hope is a fair account of the opportunities and challenges surrounding clinical AI in the NHS. There are those who believe that AI has shown such promise that using AI to replace clinical decision making will be an irresistible solution in the face of economic and productivity challenges. Indeed, delegating clinical decision making to an algorithm not limited by working hours or fatigue might seem like a straightforward decision for those looking for a ‘quick fix’ to care delivery challenges. However, there are a raft of concerns cited by opponents, with the most commonly cited arguments being patient safety concerns and an immaturity of necessary governance mechanisms. In this special issue, the *Future Healthcare Journal* presents its second debate article. Jens Christian Berring and Anne Kinderlerer provide the perspectives of the proposition and opposition respectively, and we welcome your thoughts through our online voting system to help answer the question: *‘Will artificial intelligence replace clinical decision making within our lifetimes?’.*^20^ We look forward to reading and reflecting upon your thoughts.

## Funding sources

This research did not receive any specific grant from funding agencies in the public, commercial, or not-for-profit sectors.

## CRediT authorship contribution statement

**Anmol Arora:** Conceptualization, Writing – original draft, Writing – review & editing. **Tom Lawton:** Conceptualization, Writing – original draft, Writing – review & editing.

## Declarations of competing interest

The authors declare that they have no known competing financial interests or personal relationships that could have appeared to influence the work reported in this paper.
